# The Effects of Illegitimate Tasks on Task Crafting and Cyberloafing: The Role of Stress Mindset and Stress Appraisal

**DOI:** 10.3390/bs14070600

**Published:** 2024-07-14

**Authors:** Qian Ma, Yuxuan Xie

**Affiliations:** 1 School of Labor and Human Resources, Renmin University of China, Beijing 100872, China; 2 School of Economics and Management, Tsinghua University, Beijing 100084, China; xyx21@mails.tsinghua.edu.cn

**Keywords:** illegitimate tasks, stress mindset, cognitive appraisals, task crafting, cyberloafing

## Abstract

Previous studies have mainly focused on the detrimental effects of illegitimate tasks as ubiquitous workplace stressors while ignoring the appraisal measures for such tasks. The term “illegitimate” is used by employees to describe the alignment of a task with their job role rather than the inherent qualities of the task itself. Thus, drawing on the transactional theory of stress, this study examines the moderating effect of the stress mindset on the relationship between illegitimate tasks and the appraisal of such tasks. On this basis, this study further explores when cognitive appraisal mediates the effects of illegitimate tasks on coping behaviors (task crafting and cyberloafing). Data were collected from 285 employees from an energy company in Shandong, China, by using a time-lagged research design. The findings indicate that employees react differently to illegitimate tasks depending on their stress mindset. Specifically, for employees with a stress-is-enhancing mindset, illegitimate tasks induce their challenge appraisal, which leads to task crafting. In addition, illegitimate tasks induce hindrance appraisal in employees with a stress-is-debilitating mindset, which leads to cyberloafing. In practice, this research study suggests that when illegitimate tasks cannot be eliminated, organizations should consider employees’ stress mindset when assigning such tasks.

## 1. Introduction

Illegitimate tasks are task-related stressors and are defined as tasks that go against the norms of what an employee reasonably expects to do, encompassing unreasonable and unnecessary tasks [1]. Unreasonable tasks are beyond the employee’s occupational role or status and may put them in an uncomfortable or difficult situation. Unnecessary tasks are tasks that could have been avoided or should not exist [1,2]. Illegitimate tasks convey social signals of disrespect, disregard, or devaluation, posing a threat to the employee’s self-view [2]. Previous studies primarily focused on the detrimental effects of illegitimate tasks. Specifically, task illegitimacy has destructive effects on employee feelings, work attitudes, and behaviors, such as self-esteem, job satisfaction, emotions (e.g., anger), well-being, and proactive work behavior [2,3,4,5,6,7,8,9].

However, the term “illegitimate” reflects an employee’s appraisal of how well a task aligns with their job responsibilities rather than the inherent qualities of the task itself [2,4]. In some instances, tasks are perceived as illegitimate because they are beyond the employee’s competence. Even a highly prestigious task can be perceived as illegitimate if it exceeds the employee’s job scope, for example, requesting that a newcomer familiarize themselves with advanced operation systems that are not currently used. As indicated in previous research, the perception of task stressors among individuals is inherently subjective [10]. This suggests that not all employees appraise illegitimate tasks as hindering personal growth, leading to potential differences in reactions when assigned such tasks. Therefore, a gap exists in prior research, as it has not fully considered the appraisal measures for illegitimate tasks. Moreover, given the diverse ways in which employees appraise these tasks, it prompts the question of whether employees invariably respond negatively to such tasks.

To fill this gap, this study examines when and why employees differ in their reactions to illegitimate tasks. Given the variations in how individuals interpret these tasks, it is crucial to consider the boundary conditions of individual differences in the relationship between illegitimate tasks and subsequent outcomes. Drawing on the transactional theory of stress, we investigate the conditions under which illegitimate tasks can be conducive to personal growth and unpack the mechanisms through which illegitimate tasks affect employee behaviors. Illegitimate tasks can be a source of workplace stress because they offend individuals’ professional identity and threaten their self-view [2]. According to the transactional theory of stress, employees may appraise a singular stressor as either a challenge or a threat and engage in corresponding coping behaviors [11,12]. Individuals’ evaluation of a stressor is contingent upon its interaction with their stable characteristics. Individuals may appraise the same stressor differently, and these appraisals can affect how they respond to stress [11]. In this study, we introduce an important personality trait, the stress mindset, to explain why employees respond differently to illegitimate tasks. Stress mindset refers to the extent to which an individual believes that stress has either enhancing or debilitating consequences for stress-related outcomes such as personal growth, performance, and well-being [13]. Those who believe stress improves these outcomes hold a “stress-is-enhancing mindset”, while those who believe stress hinders these outcomes hold a “stress-is-debilitating mindset”. Individual mindsets serve to evaluate demanding situations that significantly affect how the individual perceives and responds to these demands. Additionally, previous research has found that individuals’ stress mindset affects their appraisal of stressful situations as well as the subsequent behavioral response [14,15,16]. Thus, the stress mindset might shape the cognitive appraisal of illegitimate tasks and thus indirectly affect the coping behavior generated by the appraisal of illegitimate tasks. To further explore the behavioral response, task crafting and cyberloafing were selected as representative variables of approach and avoidance coping behaviors.

The most significant contribution of this study is that it considers the appraisal measures for illegitimate tasks, especially the role of the stress mindset in explaining why employees respond differently to illegitimate tasks. We answer calls for research on the stress appraisal of illegitimate tasks [3,6]. This research study goes beyond previous study, which tested only the subdimension of overall illegitimate tasks, unreasonable tasks, and ignored how individual differences mediated primary appraisals [17]. Additionally, this research study provides empirical support for the notion that individual differences exert influence when hindrance stressors are appraised as challenging [18]. Such an understanding is important for supervisors when using personality measures to screen employees who will face illegitimate tasks.

Second, drawing upon the transactional theory of stress, we demonstrate the mediating role of cognitive appraisal and provide a cognitive perspective to explain the relationship between illegitimate tasks and different coping behaviors, task crafting, and cyberloafing. Although previous studies have provided various explanations for the effects of illegitimate tasks, such as role theory [10,19] and stress-as-offense-to-self theory [4], the cognitive perspective employed in this research study allows for a deeper exploration of the double-edged effects of illegitimate tasks. It also illuminates the sense-making processes that influence employees’ responses to such tasks.

Finally, this study contributes to the existing literature on illegitimate tasks by providing a more comprehensive perspective on their effects. This study contends that illegitimate tasks can prompt proactive behaviors via challenge appraisal depending on employees’ stress mindset, which responds to calls for research into the bright side of illegitimate tasks [3].

To summarize, this study aims to develop a dual-pathway model to illustrate how employees’ cognitive appraisals and coping behaviors are influenced by illegitimate tasks, contingent upon their stress mindset. Specifically, it seeks to examine the effect of the interaction between illegitimate tasks and the stress mindset on employees’ cognitive appraisal. Furthermore, it also explores how varying beliefs about stress can influence the interpretation of illegitimate tasks and subsequent coping strategies.

## 2. Theoretical Background and Hypotheses

### 2.1. Interactive Effects of Illegitimate Tasks and Stress Mindset on Cognitive Appraisal

The transactional theory of stress suggests that certain individual traits critically influence the cognitive appraisal process, determining the extent to which a stressor is appraised as promoting or thwarting elements of inherent value to the individual [11]. In a nutshell, differences in personality can lead to interindividual variations in stressor appraisal relationships [20,21,22]. For example, compared with low-trait-resilient employees, employees with high trait resilience are more inclined to appraise the performance pressures as challenging [20]. This study posits that illegitimate tasks and employees’ stress mindset interact to predict cognitive appraisal of illegitimate tasks.

Several studies on stress have identified the important role of the stress mindset as an individual characteristic in the stress process [13,14,16,23,24,25,26]. Stress mindset refers to the general belief that stress is either destructive or beneficial to health, growth, and productivity and critically affects the employee’s reaction to stressors. The stress mindset is a “meta-cognitive belief” regarding the nature of stress [13]. Unlike stress appraisal, which is context-dependent, the stress mindset represents a general and domain-agnostic simplifying system that is not specific to any particular situation [27]. Individuals with a stress-is-enhancing mindset are inclined to concentrate on the positive information from stressors and reinforce the positive beliefs, resulting in appraising stressors as challenges and employing active coping strategies. In contrast, employees with a stress-is-debilitating mindset are inclined to appraise stressors as inhibitive and taxing, leading them to adopt avoidance behaviors as a coping strategy.

We propose that employees’ appraisal of illegitimate tasks may vary depending on their stress mindset. Illegitimate tasks are work stressors that not only undermine individuals’ professional identity but also threaten their self-view, potentially leading to feelings of worthlessness and disrespect [2]. However, the perception of a task as illegitimate does not necessarily stem from its demeaning nature [10]; rather, employees’ perceptions of task legitimacy are subjective. As previous research indicates, the legitimacy of a task is not inherently determined but depends on the individual involved [1,10,28]. While certain tasks may fall outside one’s designated occupational responsibilities, they can still present challenges and contribute to one’s sense of competence [3,29]. We hypothesize that employees with a stress-is-enhancing mindset are inclined to perceive illegitimate tasks as a challenge compared with those with a stress-is-debilitating mindset. Since employees with a stress-is-enhancing mindset believe in the enhancing properties of stressors, they tend to regard the additional work expectations as opportunities for learning (e.g., acquiring new experiences) and growth (e.g., fostering job variety and getting recognition from leaders). They focus on the potential benefits of overcoming such tasks [30]. For example, accomplishing such tasks may prove their competence and build a positive relationship with leaders. In contrast, employees with a negative stress belief are inclined to concentrate on the passive aspects of illegitimate tasks. Illegitimate tasks represent inappropriate task allocation, thereby violating the norms associated with the fundamental requirements of the employee’s profession [1]. Furthermore, undertaking illegitimate tasks is likely to signal social exclusion, leading employees to experience feelings of disrespect and unfairness [31]. As employees focus on these adverse impacts of illegitimate tasks, the information reinforces their negative beliefs about illegitimate tasks, leading them to appraise the task as a hindrance. Thus, we form the following hypotheses:

**Hypothesis** **1a.** Illegitimate tasks and stress mindset interact to influence challenge appraisal of illegitimate tasks such that the relationship is more positive for employees with a stress-is-enhancing mindset.

**Hypothesis** **1b.** Illegitimate tasks and stress mindset interact to influence hindrance appraisal of illegitimate tasks such that the relationship is more positive for employees with a stress-is-debilitating mindset.

### 2.2. Primary Appraisals and Secondary Appraisals

In the primary appraisal step, employees appraise illegitimate tasks as either a challenge or a hindrance, depending on their stress mindset. Based on the transactional theory of stress, the primary appraisal stimulates subsequent actions to cope with stressors [11,32,33]. Multiple classifications have been established to categorize coping strategies; the one that aims to address or avoid the demand is the approach–avoidance coping strategy [34]. Previous research has shown that individuals may cope with stressors by actively approaching the situation or by avoiding it. When stressors are perceived as challenging, individuals are more inclined to engage in functional behaviors; for stressors perceived as hindrances, individuals are more prone to exhibit dysfunctional behaviors [35].

Challenge appraisals indicate that the employee considers the current situation as conducive to personal growth and goal attainment [36]. Therefore, challenge appraisals trigger approach-oriented coping tendencies, whereby individuals actively engage with the situation through bursts of inspiration and high levels of effort [37]. Previous research has found that challenge appraisals correlate positively with problem-focused coping strategies, approach-oriented coping tendencies, and proactive behavior [38,39,40].

Consistently with previous research, we propose that when employees perceive illegitimate tasks as challenges, they are more inclined to proactively address the situation. This may involve modifying their work tasks and roles to achieve their work goals, a concept known as task crafting. Job crafting, a form of proactive behavior, involves employees taking voluntary actions to reshape, modify, and redefine their job in order to enhance their work experience [41]. Task crafting is the core behavioral component of job crafting, which aims to change the scope of tasks included in the job.

According to the proactive motivation model, three motivational states (“can do”, “reason to”, and “energized to”) have been identified to drive proactive behavior [42]. Challenge appraisal may encourage task crafting via the “reason to” and “energized to” pathways. Challenge appraisal indicates that the current situation is promotive and facilitative and assumes that addressing the situation will likely produce positive outcomes, providing “reason to” invest time and effort to engage in proactive behavior [40]. On the other hand, challenge appraisal can stimulate positive effects and lead to increased motivation [36,43], providing energy for employees to address these demands proactively. Task crafting, which involves employees making proactive changes to improve their work, is an approach coping strategy [44]. Empirical studies have demonstrated that a positive relationship exists between challenge appraisal and job crafting. Specifically, research indicates that individuals who view stress as a challenge are more inclined to engage in promotion-focused job crafting behavior [45]. When stress is viewed as a challenge, employees are motivated to seek proactive strategies such as job crafting to cope with challenging demands [46]. On this basis, challenge appraisal should be positively related to task crafting. Thus, we propose the following:

**Hypothesis** **2a.** Challenge appraisal is positively related to task crafting.

Conversely, hindrance appraisal indicates that the demand harms the employee’s personal development and thwarts goal accomplishment. When employees encounter obstacles in pursuing self-relevant work goals, they may psychologically disengage from the situation and exhibit diminished levels of work motivation [37,47,48]. Hindrance appraisal elicits more avoidance or emotion-focused coping behaviors [47]; for example, hindrance appraisal is positively related to turnover intentions [12], procrastination behavior [49], and negative proactive behavior [39].

The convenience and concealment of cyberloafing, make it an effective withdrawal coping strategy [50]. We thus propose that hindrance appraisal promotes employee cyberloafing activities. Cyberloafing is defined as the use by employees of the internet for personal activities during work hours. This includes non-work-related tasks such as sending emails, online shopping, and browsing unrelated websites [51]. Employees may pretend to be working, and cyberloafing is hardly detected by supervisors and coworkers. Previous studies show that cyberloafing is a prominent form of avoidance or emotion-focused coping strategy that helps employees cope with work-related stressors (e.g., workplace boredom, job burnout, and work aggression) [52,53,54]. Specifically, cyberloafing may serve as a “micro-break”, allowing employees to psychologically detach from such stressful tasks and benefit from recovery effects [55,56,57]. Thus, employees with hindrance appraisal are more likely to distance themselves from their tasks and seek immediate gratification though cyberloafing. We, therefore, propose the following:

**Hypothesis** **2b.** Hindrance appraisal is positively related to employee cyberloafing.

### 2.3. Moderated Mediating Effects

Taken together, by affecting illegitimate task–stress appraisal relationships, the stress mindset further moderates indirect relationships between illegitimate tasks and employees’ coping behaviors via cognitive appraisal. On the one hand, employees with a stress-is-enhancing mindset are inclined to view illegitimate tasks as challenging, leading to increased approach-coping behaviors compared with those with a stress-is-debilitating mindset. When faced with illegitimate tasks, employees with a stress-is-enhancing mindset are inclined to perceive these tasks as opportunities to overcome challenges and achieve rewards and growth. Additionally, if employees view these tasks as opportunities for future benefits and advancement, they are inspired to proactively cope with these demanding situations. This may involve making changes to work processes or taking control over their tasks.

On the other hand, employees who hold a stress-is-debilitating mindset tend to view illegitimate tasks as hindrances and react passively, performing activities such as cyberloafing. Illegitimate tasks can be seen as a threat to professional identity and self-esteem, leading those with a stress-is-debilitating mindset to concentrate on the detrimental effects of such tasks and appraise those tasks as hindering personal development and well-being. In turn, employees are more likely to disengage from such problems by engaging in cyberloafing. Therefore, we contend that a stress-is-debilitating mindset amplifies the mediating role of hindrance appraisal in the relationship between illegitimate tasks and cyberloafing. Thus, we propose the following:

**Hypothesis** **3a.** The stress mindset moderates the indirect relationship between illegitimate tasks and task crafting via challenge appraisal, such that the relationship is stronger for employees with a stress-is-enhancing mindset.

**Hypothesis** **3b.** The stress mindset moderates the indirect relationship between illegitimate tasks and cyberloafing via hindrance appraisal, such that the relationship is stronger for employees with a stress-is-debilitating mindset.

**Figure 1:**
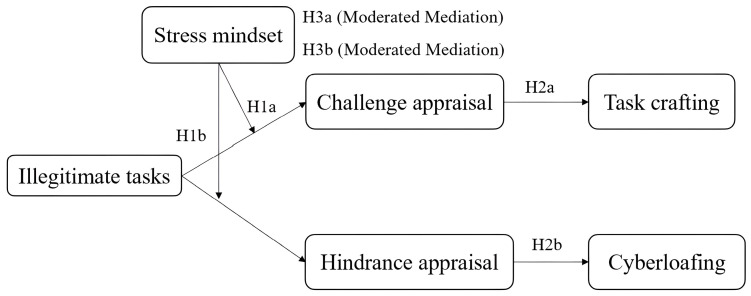
Conceptual model.

The conceptual model is shown in Figure 1.

## 3. Materials and Methods

### 3.1. Sample and Procedure

This study used a sample of 285 participants working in an energy company in Shandong province, China. We collected data through an online survey platform (https://www.wjx.cn, accessed on 21 May 2023). To control for common-method variance, data were collected at two different points in time, one month apart. At Time 1, 460 participants provided information on their demographic details, illegitimate tasks, stress mindset, and challenge and hindrance appraisal. To ensure data quality, we implemented pre-existing safeguards, including two attention check items. We screened out 54 invalid questionnaires that did not meet specific criteria, such as incorrect answers to the attention check questions or unusually short or long response times. Subsequently, at Time 2, 335 of these participants (72.83%) reported on task crafting and cyberloafing. By aligning the responses recorded at the two time points and conducting data cleansing based on the same criteria, we obtained a final sample of 285 participants with a response rate of 61.96%. Participants received CNY 20 as compensation upon completion of both surveys, and their cellphone’s last four digits were used to track and match records from both time points. We emphasized that the data collected were solely for academic research purposes, and the entire process was conducted anonymously. All participants provided their consent for the study.

Males made up 41.1% of the respondents, and 75.4% of the respondents had a bachelor’s degree. Their average age was 31 years [ranging from 22 to 59; standard deviation (SD) = 6.936]. The average employment period in their current organization was 7.80 years (SD = 6.779).

### 3.2. Measures

Given that the original scales measuring focal variables in this study were in English, we employed Brislin’s (1986) [58] standardized translation and back-translation procedures to develop Chinese versions of these scales. All variables were measured on a five-point Likert scale. For the measurement items used in this study, see Appendix A.

Illegitimate tasks. The Bern Illegitimate Task Scale comprises eight items, consisting of four unreasonable task items and four unnecessary task items [7]. Sample items are “Do you have work tasks to take care of which you believe put you into an awkward position?” (i.e., unreasonable tasks) and “Do you have work tasks to take care of which keep you wondering if they make sense at all?” (i.e., unnecessary tasks). Cronbach’s alpha was 0.88.

Stress mindset. We assessed the stress mindset by using the eight-item measure developed by Crum et al. (2013) [13]. Participants rated how strongly they agreed with the statements. Sample items are “Experiencing stress facilitates my learning and growth” and “Experiencing stress depletes my health and growth” (reverse scored). Higher scores represent the mindset that the effects of stress are enhancing. Cronbach’s alpha was 0.90.

Challenge and hindrance appraisal. We used the instrument by LePine et al. (2016) [40] to measure challenge and hindrance appraisal with three items each. Items included “In general, I feel that my job promotes my personal accomplishment.” (i.e., challenge appraisal, *α* = 0.61) and “I feel the demands of my job constrain my achievement of personal goals and development.” (i.e., hindrance appraisal, *α* = 0.67).

Task crafting. Task crafting was measured by using five items developed by Slemp and Vella-Brodrick (2013) [59]. Participants rated the frequency with which they engaged in each task-crafting activity; an example of an item is “Choose to take on additional tasks at work.” Cronbach’s alpha was 0.71.

Cyberloafing. Cyberloafing was assessed by using the five-item scale by Zoghbi-Manrique-de-Lara, Verano-Tacoronte, and Ding (2006) [60] adapted from that proposed by Lim (2002) [51]. An example of an item is “I visit websites and digital newspaper to seek personal information.” Cronbach’s alpha was 0.82.

Control variables. Following prior research [4,8], we controlled for age, gender, and education. Gender was coded as follows: male = 1 and female = 2. Education was coded as follows: below college degree = 1, college degree = 2, bachelor’s degree = 3, master’s degree = 4, and doctorate degree = 5. Employee age was measured in years. Job tenure was not included in the control variables in this study because it correlated strongly with employee age (*r* = 0.969, *p* < 0.001).

### 3.3. Statistical Analysis Methods

To test the hypotheses, we used path analysis and estimated all path parameters simultaneously with Mplus 8 [61]. The predictors (i.e., illegitimate tasks and stress mindset) were centered to reduce estimation and multicollinearity problems. We followed previous procedures to test the moderating effects [62]. The moderated mediation hypotheses were tested by estimating the index of moderated mediation and calculating the condition indirect effects (−1 SD, mean, and +1 SD) through the mediator. Confidence intervals (CIs) were estimated at 95% based on data from 5000 bootstrapped samples to assess the significance of the hypothesized moderated mediating relationships.

## 4. Results

### 4.1. Confirmatory Factor Analysis

Before hypothesis testing, a series of confirmatory factor analyses were conducted to assess the distinctiveness of the hypothesized six-factor model. The results presented in Table 1 indicate that compared with alternative models, the proposed six-factor model produces an acceptable fit to the data (χ^2^ = 830.620, χ^2^/df = 1.850, CFI = 0.904, TLI = 0.894, RMSEA = 0.055, and SRMR = 0.056). Although TLI is slightly less than 0.90, the six-factor model based on a two-index presentation strategy produces an adequate fit [8,63]. Specifically, a model with relatively good fit would be evidenced by an SRMR value of close to 0.08 (or less) and an RMSEA value of close to 0.06 (or less) [64]. Thus, the six-factor model exhibited good discriminant validity in this study.

Self-reported data from employees in this study were analyzed by using Harman’s single-factor test to evaluate common-method variance [65]. The findings indicate that the first factor explains 29.32% of the total variance, which is less than the critical threshold of 40%, suggesting that common-method variance does not significantly affect the results of this study.

### 4.2. Descriptive Statistics and Correlation Analysis

Table 2 displays the descriptive information and correlation coefficients of the variables. The findings indicate that illegitimate tasks correlated negatively with challenge appraisal (*r* = −0.274, *p* < 0.001), stress mindset (*r* = −0.333, *p* < 0.001), and task crafting (*r* = −0.313, *p* < 0.001) but positively with hindrance appraisal (*r* = 0.337, *p* < 0.001) and cyberloafing (*r* = 0.483, *p* < 0.001). Challenge appraisal correlated positively with task crafting (*r* = 0.525, *p* < 0.001), and hindrance appraisal correlated positively with cyberloafing (*r* = 0.361, *p* < 0.001).

### 4.3. Testing the Hypotheses

#### 4.3.1. Moderating Effects

**Figure 2:**
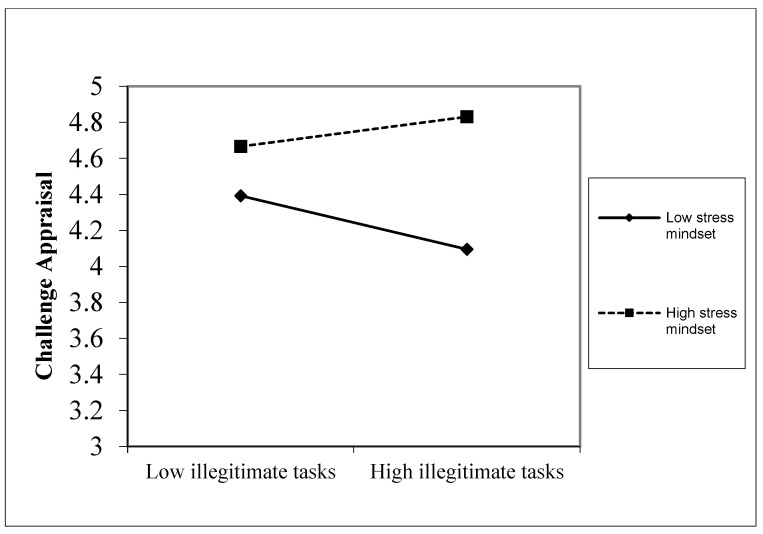
Interactive effects of illegitimate tasks and stress mindset on challenge appraisal.

The results presented in Table 3 and Table 4 demonstrate a positive and significant impact of the interaction between illegitimate tasks and stress mindset on challenge appraisal (*b* = 0.223, *p* < 0.001). Figure 2 depicts the interaction plot that shows that illegitimate tasks positively predicted challenge appraisal for employees with a stress-is-enhancing mindset (+1 SD) (*b* = 0.116, *p* < 0.01). Conversely, for employees with a stress-is-debilitating mindset (−1 SD), illegitimate tasks significantly and negatively predicted challenge appraisal (*b* = −0.208, *p* < 0.01). Thus, hypothesis 1a is supported.

**Figure 3:**
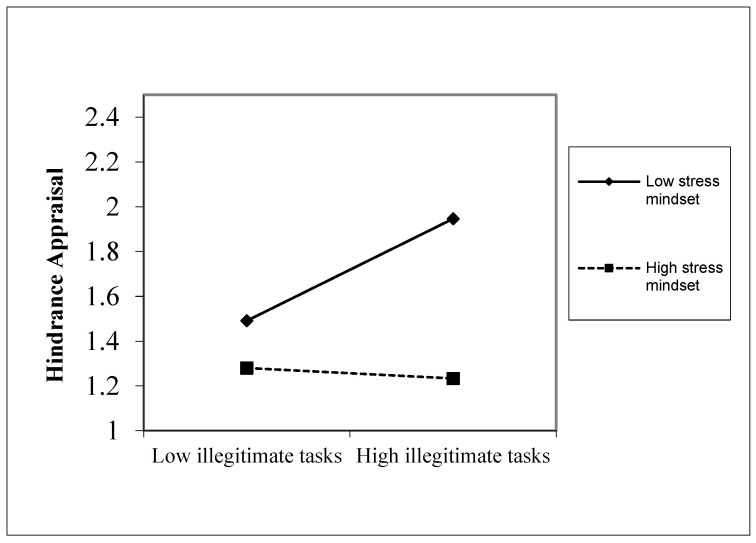
Interactive effects of illegitimate tasks and stress mindset on hindrance appraisal.

Hypothesis 1b states that illegitimate tasks and employees’ stress mindset interact to influence employees’ hindrance appraisal of illegitimate tasks. The results listed in Table 3, Model 6 show that the effect of the interaction between illegitimate tasks and stress mindset on hindrance appraisals was negative and significant (*b* = −0.245, *p* < 0.001). Simple slope analysis indicates that for employees who view stress as beneficial, there was no connection between illegitimate tasks and hindrance appraisal (*b* = −0.047, *ns*). However, for employees with a stress-is-debilitating mindset, illegitimate tasks positively predicted hindrance appraisal (*b* = 0.309, *p* < 0.001) (Figure 3). Therefore, hypothesis 1b is supported.

#### 4.3.2. Direct Effects Test

The results listed in Table 3 show that consistent with our prediction, a positive correlation existed between challenge appraisal and task crafting (*b* = 0.577, *p* < 0.001), as well as a positive correlation between hindrance appraisal and cyberloafing (*b* = 0.266, *p* <0.01). Thus, hypotheses 2a and 2b are supported.

#### 4.3.3. Moderated Mediation Analysis

Hypotheses 3a and 3b predict that the stress mindset moderates the indirect relationship between illegitimate tasks and coping behaviors via cognitive appraisal. Hypotheses 3a and 3b predict that the stress mindset moderates the indirect relationship between illegitimate tasks and coping behaviors via cognitive appraisal. The results listed in Table 5 show a significant index of moderated mediation (estimate = 0.187, SE = 0.042, 95% CI [0.106, 0.250]). Specifically, for employees with a stress-is-enhancing mindset, the indirect effect of illegitimate tasks on task crafting via challenge appraisal was positive (estimate = 0.067, SE = 0.022, 95% CI [0.030, 0.116]). For employees with a stress-is-debilitating mindset, the indirect effect of illegitimate tasks on task crafting via challenge appraisal was negative (estimate = −0.120, SE = 0.034, 95% CI [−0.176, −0.053]). Thus, hypothesis 3a is supported.

For hypothesis 3b, the simple indirect effect of illegitimate tasks on cyberloafing via hindrance appraisal was significant and positive for employees with a stress-is-debilitating mindset (−1 SD: estimate = 0.082, SE = 0.033, 95% CI [0.029, 0.162]) but nonsignificant for employees with a stress-is-enhancing mindset (+1 SD: estimate = −0.012, SE = 0.015, 95% CI [−0.048, 0.013]).The difference between these indirect effects was significant (estimate = −0.095, SE = 0.038, 95% CI [−0.183, −0.031]), providing support for Hypothesis 3b.

## 5. Discussion

Drawing on the transactional theory of stress, this study theorized about the variation in employees’ responses to illegitimate tasks, with a specific focus on the role of stress mindset in the stress process.

First, the present study revealed that how employees appraise illegitimate tasks is influenced by their stress mindset. Specifically, employees with a stress-is-enhancing mindset are more likely to appraise illegitimate tasks as challenging. In contrast, employees with a stress-is-debilitating mindset are more likely to appraise illegitimate tasks as hindering. The findings are consistent with previous studies that emphasize the promoting nature of illegitimate tasks [1,29,66,67]. Moreover, in line with existing research, the findings indicate that the stress mindset plays an important role in determining whether individuals experience workplace stressors as challenges or hindrances [14].

Second, the findings support that challenge appraisal mainly affected approach coping behavior (e.g., task crafting), whereas hindrance appraisal mainly affected avoidance coping behavior (e.g., cyberloafing). These findings align with previous studies indicating that individuals are more likely to engage in adaptive behaviors when they perceive stressors as challenges. Conversely, when stressors are perceived as hindrances, individuals are more prone to exhibit maladaptive behaviors [39,49].

Finally, the findings suggest that illegitimate tasks affect employees’ cognitive appraisals and that the subsequent coping behaviors depend on employees’ stress mindset. Specifically, employees with a stress-is-enhancing mindset perceive illegitimate tasks as challenges and engage in task crafting. In contrast, illegitimate tasks induce hindrance appraisals for those with a stress-is-debilitating mindset and lead to cyberloafing. On the one hand, building on previous studies that have shown employees resorting to avoidance coping strategies when faced with illegitimate tasks, such as turnover intentions and counterproductive workplace behavior (CWB) [31,68], our research suggests that employees may also turn to cyberloafing as a way to cope with illegitimate tasks. Furthermore, our findings are consistent with prior research highlighting the adverse effects of illegitimate tasks, indicating that they can be considered hindrance stressors that impede personal development and work-related achievements. On the other hand, in contrast with the existing literature that predominantly focuses on the negative impacts of illegitimate tasks, this study sheds light on the potential bright side of such tasks. Employees with a positive mindset may focus on the promoting nature of illegitimate tasks and engage in task crafting.

Overall, these findings highlight the significance of considering cognitive appraisal and the stress mindset in studying the impacts of illegitimate tasks. They emphasize the importance of employees’ stress mindset in shaping their experiences and underscore why employees have different reactions to illegitimate tasks.

### 5.1. Theoretical Contributions

This study contributes to the existing research on illegitimate tasks by introducing employees’ stress mindset as a significant moderator for primary appraisal of illegitimate tasks. These results enhance our understanding of the varying reactions that individuals have to illegitimate tasks and suggest that illegitimate tasks correlate positively with challenge appraisal in employees with a stress-is-enhancing mindset, whereas employees with a stress-is debilitating mindset tend to perceive illegitimate tasks as hindrances. Although previous studies indicated that an individual’s sense of control over a situation, such as trait resilience and self-esteem, can impact the cognitive appraisal process, these characteristics do not fully elucidate how individuals may perceive and interpret demands [4,20]. Thus, this study also enriches the stress mindset literature by revealing that the stress mindset determines how individuals appraise stressors and choose coping strategies when confronted with illegitimate tasks. This study thus unpacks the relationships among workplace stressors, stress appraisals, and stress mindset [23]. Moreover, studying the stress mindset as the boundary condition not only enriches the knowledge of individual differences in the primary appraisal of work stressors (e.g., illegitimate tasks in this study) but also provides a clear account of when primary appraisal operates to intervene in the effects of work stressors on employee outcomes. Our findings can also be explained by trait activation theory, which posits that individual traits have latent potential that influences how individuals behave, think, and feel in distinct ways based on situational trait-relevant cues [69]. In this study, the impact of the stress mindset was significant in high-demand situations. Whether employees have positive or negative beliefs about stress shapes their reactions to illegitimate tasks.

Second, drawing upon the transactional theory of stress, this research study revealed the mechanism by which illegitimate tasks trigger different coping behaviors from the perspective of cognitive appraisal. Previous research overlooked the importance of stressor appraisal in the context of illegitimate tasks, whereas this study addresses that gap by highlighting stress appraisal as the key factor in understanding how illegitimate tasks influence subsequent behaviors. Thus, our results prove the study which shows that a single stressor can be appraised simultaneously as challenging and hindering [12]. Moreover, the results respond to the call for considering the appraisal measures for illegitimate tasks [3,6].

Finally, in contrast with past studies that exclusively concentrated on the deleterious effects of illegitimate tasks, this study answers calls for research on the bright side of illegitimate tasks [3]. The results demonstrate that not all employees react with an avoidance coping strategy when assigned illegitimate tasks. Employees who hold a positive stress mindset tend to appraise illegitimate tasks as challenges and employ approach coping mechanisms, such as task crafting, to effectively manage the situation. Despite previous studies having explored the buffering role of job crafting in the relationship between illegitimate tasks and their outcomes (e.g., meaning of work and health problems) [70,71], this research study adds new insights to investigate how illegitimate tasks impact job crafting. In addition, responding to the call for investigation into more contextual and motivational antecedents of job crafting, our study introduced individual appraisal as the antecedent [72]. Unsurprisingly, this study revealed that “victims” of illegitimate tasks reacted by spending time on the internet for non-work-related use, which aligns with prior research on the negative effects of illegitimate tasks on other outcomes, confirming that these job demands are stressors at work [8,31]. Although prior studies have shown that illegitimate tasks can result in CWB [7,31,73], they largely encompassed general CWB, except for destructive voice and time theft [74]. This study focuses on the specific manifestations of CWB, e.g., cyberloafing, which enriches research on illegitimate tasks and cyberloafing.

### 5.2. Practical Implications

The present results have important practical implications. First, and maybe most importantly, this research study highlights the crucial impact of employees’ stress mindset on their reactions to illegitimate tasks. Given these results, illegitimate tasks induce challenge appraisal among employees with a stress-is-enhancing mindset, prompting task crafting. In contrast, employees with a stress-is-debilitating mindset are more likely to perceive illegitimate tasks as a hindrance, resulting in avoidance coping behavior, cyberloafing in this study. Since illegitimate task requests are sometimes unavoidable, these findings emphasize that managers should consider employees’ stress mindset when assigning such tasks. On the one hand, managers could use the stress mindset as a selection criterion to prioritize candidates with a stress-is-enhancing mindset. On the other hand, like other mindsets, the stress mindset can be easily manipulated. Stress mindset interventions focus on changing individuals’ construal of stress. By implementing interventions, participants may realize that they can leverage the stress response for positive outcomes [75,76]. Previous research showed that individuals’ stress mindset can be altered through video interventions [13,77]. After a “stress-can-be-enhancing mindset” intervention, participants reported better performance, improved health, elevated positive effects, and improved cognitive functioning [13,77,78,79]. Thus, organizations may consider providing live training on stress mindsets to help employees rethink stress. Having a healthy stress mindset may enable employees to enhance their resilience and effectively engage with stress.

Second, the negative effects of illegitimate tasks cannot be overlooked. Employees with a stress-is-debilitating mindset tend to engage in non-work-related activities via hindrance appraisal when confronted with illegitimate tasks. Therefore, organizations should implement effective measures to reduce the occurrence of illegitimate tasks. For example, organizations may adopt artificial intelligence to simplify tasks and eliminate necessary tasks. Additionally, supervisors can help mitigate the detrimental effects of illegitimate tasks by emphasizing the importance of tasks or recognizing the contributions of employees when assigning such tasks [68].

### 5.3. Limitations and Directions for Future Research

This study has several limitations. First, all variables were self-reported, raising concerns about common-method bias. However, illegitimate tasks, stress mindset, and cognitive appraisal inherently rely on self-reported measures because they assess internal phenomena related to self-conception and perception. Furthermore, we followed the suggestion by Podsakoff et al. (2003) [65] and collected data at different time points. Nevertheless, future studies could benefit from incorporating objective measures of cyberloafing, such as monitoring employee’s internet usage in experimental settings, to complement our self-reported measures. Second, given that all relationships reported herein are correlational, causal influences cannot be inferred. Third, the samples in this study were collected in a Chinese cultural context, where employees value collectivism [80]. Previous studies have found that employees from highly collectivistic cultures may be more tolerant of illegitimate tasks [81]. Thus, future research should explore the moderating role of cultural values. Moreover, to validate the conclusions, further research should include participants from different countries and various work contexts. Finally, this study only examines the moderating effect of individual differences. Future research should examine the impact of other contextual and contingency factors (e.g., workload and time pressure) in the appraisal process.

## 6. Conclusions

This research study enriches the literature on illegitimate tasks by examining the appraisal measures for illegitimate tasks and their potential positive impacts. Drawing on the transactional theory of stress, the results indicate that employees’ stress mindset plays a significant role in determining their responses to illegitimate tasks. In practice, these findings emphasize that when illegitimate tasks cannot be eliminated, organizations should consider providing live training on stress mindsets to assist employees in reframing stress and fostering a stress-is-enhancing mindset.

## Figures and Tables

**Table 1:** Results of confirmatory factor analysis.

Models	χ^2^	df	χ^2^/df	RMSEA	SRMR	CFI	TLI
Six-factor model (IT, SM, CA, HA, TC, and CY)	830.620	449	1.850	0.055	0.056	0.904	0.894
Five-factor model (IT, SM, CA + HA, TC, and CY)	858.477	454	1.891	0.056	0.058	0.898	0.889
Five-factor model (IT, SM, CA, HA, and TC + CY)	1076.830	454	2.372	0.069	0.074	0.843	0.829
Five-factor model (IT + SM, CA, HA, CY, and TC)	1626.906	454	3.583	0.095	0.107	0.705	0.678
Four-factor model (IT + SM, CA + HA, TC, and CY)	1650.336	458	3.603	0.096	0.107	0.700	0.675
Three-factor model (IT + SM, CA + TC, and HA + CY)	1887.003	461	4.093	0.104	0.120	0.641	0.614
Two-factor model (IT + SM + CA + HA, and TC + CY)	2022.411	463	4.368	0.109	0.116	0.608	0.580
One-factor model (IT + SM + CA + HA + TC + CY)	2249.619	464	4.848	0.116	0.114	0.551	0.520

Notes: IT = illegitimate tasks; SM = stress mindset; CA = challenge appraisal; HA = hindrance appraisal; TC = task crafting; CY = cyberloafing. “+” means two factors merged into one factor.

**Table 2:** Means, standard deviations, and correlations of study variables.

Variables	1	2	3	4	5	6	7	8	9
1. Gender	-								
2. Age	−0.026	-							
3. Education	−0.054	−0.163 **	-						
4. Illegitimate tasks	0.019	−0.223 ***	−0.016	-					
5. Stress mindset	−0.109	0.151 *	0.015	−0.333 ***	-				
6. Challenge appraisal	−0.092	0.098	−0.046	−0.274 ***	0.582 ***	-			
7. Hindrance appraisal	0.054	−0.134 *	0.087	0.337 ***	−0.495 ***	−0.698 ***	-		
8. Task crafting	−0.042	0.122 *	0.094	−0.313 ***	0.509 ***	0.525 ***	−0.398 ***	-	
9. Cyberloafing	0.050	−0.268 ***	−0.025	0.483 ***	−0.292 ***	−0.316 ***	0.361 ***	−0.375 ***	-
*Mean*	1.59	31.00	3.03	2.45	3.84	4.31	1.70	3.68	2.17
*SD*	0.49	6.94	0.57	0.72	0.72	0.52	0.62	0.63	0.68

Notes: N = 285, * *p* < 0.05, ** *p* < 0.01, and *** *p* < 0.001.

**Table 3:** Results of path analysis.

	Challenge Appraisal		Hindrance Appraisal		Task Crafting		Cyberloafing	
Variables	Estimate	*SE*	Estimate	*SE*	Estimate	*SE*	Estimate	*SE*
Intercepts	4.496 ***	0.153	1.885 ***	0.163	0.669	0.438	1.885 ***	0.163
*Controls*								
Gender	−0.039	0.043	−0.026	0.059	0.009	0.062	0.083	0.067
Age	0.000	0.003	−0.007 *	0.003	0.004	0.005	−0.01 *	0.005
Education	−0.027	0.028	0.012	0.038	0.124 *	0.056	0.002	0.053
*Independent variable*								
Illegitimate tasks	−0.046	0.034	0.131 **	0.043	−0.148 **	0.050	0.353 ***	0.057
*Mediators*								
Challenge appraisal					0.577 ***	0.075		
Hindrance appraisal							0.266 **	0.085
*Moderator*								
Stress mindset	0.349 ***	0.057	−0.318 ***	0.067				
*Interaction*								
Illegitimate tasks * Stress mindset	0.223 ***	0.056	−0.245 ***	0.065				
R^2^	0.149 ***	0.028	0.246 ***	0.042	0.265 ***	0.021	0.323 ***	0.040

Notes: N = 285; unstandardized coefficients reported. SE refers to standard errors. * *p* < 0.05, ** *p *< 0.01, and *** *p *< 0.001.

**Table 4:** Results of path analysis: estimated interaction effects.

Path	Estimate	SE	Bootstrap 95% CI
Illegitimate tasks → challenge appraisal	0.223 ***	0.056	[0.088, 0.303]
High stress mindset (+1 SD)	0.116 **	0.037	[0.047, 0.191]
Low stress mindset (−1 SD)	−0.208 **	0.065	[−0.313, −0.065]
Illegitimate tasks → hindrance appraisal	−0.245 ***	0.065	[−0.341, −0.096]
High stress mindset (+1 SD)	−0.047	0.056	[−0.155, 0.062]
Low stress mindset (−1 SD)	0.309 ***	0.072	[0.154, 0.423]

Note: N = 285. Bootstrapping sample = 5000. Unstandardized coefficients reported. SE refers to standard errors. CI refers to confidence interval. ** *p* < 0.01, and *** *p* < 0.001.

**Table 5:** Results of hypothesized conditional indirect effects.

Paths	Estimate	SE	Bootstrap 95% CI
Illegitimate tasks → challenge appraisal → task crafting			
Low stress mindset (−1 SD)	−0.120 ***	0.034	[−0.176, −0.053]
Medium stress mindset	−0.027	0.019	[−0.061, 0.011]
High stress mindset (+1 SD)	0.067 **	0.022	[0.030, 0.116]
Difference (high vs. low stress mindset)	0.187 ***	0.042	[0.106, 0.250]
Illegitimate tasks → hindrance appraisal → cyberloafing			
Low stress mindset (−1 SD)	0.082 *	0.033	[0.029, 0.162]
Medium stress mindset	0.035 *	0.017	[0.009, 0.083]
High stress mindset (+1 SD)	−0.012	0.015	[−0.048, 0.013]
Difference (high vs. low stress mindset)	−0.095 *	0.038	[−0.183, −0.031]

Notes: N = 285. Bootstrapping sample = 5000. Unstandardized coefficients reported. SE refers to standard errors. CI refers to confidence interval. * *p* < 0.05, ** *p* < 0.01, and *** *p* < 0.001.

## Data Availability

The data presented in the study are available upon request from the corresponding author.

## Appendix A. Measurement

**Table A1:** The measurement items used in this study.

Scales	Items
The Bern Illegitimate Task Scale [7]	Unreasonable tasks: Do you have work tasks to take care of which you believe … 1. … should be done by someone else? 2. … are going too far and should not be expected from you? 3. …put you into an awkward position? 4. …are unfairly required of you? Unnecessary tasks: Do you have work tasks to take care of which keep you wondering if… 5. …they have to be done at all? 6. … they would not exist (or could be done with less effort), if things were organized differently? 7. …they make sense at all? 8. …they just exist because some people simply demand to have it this way?
Stress Mindset Measure [13]	1. The effects of stress are negative and should be avoided. (*Reverse*) 2. Experiencing stress facilitates my learning and growth. 3. Experiencing stress depletes my health and vitality. (*Reverse*) 4. Experiencing stress enhances my performance and productivity. 5. Experiencing stress inhibits my learning and growth. (*Reverse*) 6. Experiencing stress improves my health and vitality. 7. Experiencing stress debilitates my performance and productivity. (*Reverse*) 8. The effects of stress are positive and should be utilized.
Challenge appraisal [40]	Challenge appraisal: 1. Working to fulfill the demands of my job helps to improve my personal growth and well-being. 2. I feel the demands of my job challenge me to achieve personal goals and accomplishment. 3. In general, I feel that my job promotes my personal accomplishment. Hindrance appraisal:
Hindrance appraisal [40]	1. Working to fulfill the demands of my job thwarts my personal growth and well-being. 2. I feel the demands of my job constrain my achievement of personal goals and development. 3. In general, I feel that my job hinders my personal accomplishment.
Task crafting [59]	1. You introduce new approaches to improve your work. 2. You change the scope or types of tasks that you complete at work. 3. You introduce new work tasks that you think better suit your skills or interests. 4. You choose to take on additional tasks at work. 5. You give preference to work tasks that suit your skills or interests.
Cyberloafing [60]	I use the internet at work to… 1. Visit websites and digital newspapers to seek personal information. 2. Download software or files for personal or family use. 3. Visit the website of my bank to consult my current account. 4. Read or send personal (non-professional) emails. 5. Surf the net and so escape a little.
